# Effects of Microencapsulated Methionine on Milk Production and Manure Nitrogen Excretions of Lactating Dairy Cows

**DOI:** 10.3390/ani11123545

**Published:** 2021-12-14

**Authors:** Layla King, Janaka Wickramasinghe, Brooke Dooley, Carrie McCarthy, Emily Branstad, Ester Grilli, Lance Baumgard, Ranga Appuhamy

**Affiliations:** 1 Department of Animal Science, Iowa State University, Ames, IA 50011, USA; layla.king@ndsu.edu (L.K.); janaka@iastate.edu (J.W.); Bcdooley795@gmail.com (B.D.); Carrie.mccarthy@micro.net (C.M.); emilie@iastate.edu (E.B.); baumgard@iastate.edu (L.B.); 2 Department of Veterinary Medical Sciences, University of Bologna, 40064 Bologna, Italy; eg@vetagro.com; 3 Vetagro Inc., Chicago, IL 60604, USA

**Keywords:** digestibility of protein, environmental sustainability, mammary blood flow, rumen-protected methionine

## Abstract

**Simple Summary:**

Methionine (Met) deficiency in the diet can limit milk protein production and lead to excessive nitrogen (N) excretions to the environment by dairy cows. We demonstrated that the supplementation of a new rumen-protected Met product to a Met deficient diet increased milk protein yield and decreased manure N excretions of high producing dairy cows. Increased blood flow to the mammary glands and increased apparent total tract digestibility of dietary crude protein seem to be the underlying mechanisms for those improvements in production and the environmental sustainability.

**Abstract:**

The study objective was to determine the effects of rumen-protected methionine (Met) by microencapsulation (RPM) on amino acid (AA) supply to the udder, milk production, and manure nitrogen (N) losses of dairy cows. A corn and soybean-based diet deficient in metabolizable Met (~10 g/d) was supplemented with RPM providing 0, 11.0, 19.3, and 27.5 g/d of Met. Dry matter intake (DMI), milk production, plasma essential AA (EAA), mammary plasma flow (MPF), and fecal (FN) and urinary N (UN) outputs (g/d) were determined. The RPM increased linearly milk yield, milk protein yield, and energy corrected milk yield (*p* < 0.040) without affecting DMI. Milk protein yield increased by 50 g/d for the 19.3 vs. 0 g/d dose (*p* = 0.006) but the rate of increment decreased for 27.5 g/d dose. Plasma Met, and MPF increased linearly with RPM dose (*p* < 0.050). Apparent total tract digestibility of crude protein (*p* = 0.020) and FN (*p* = 0.081) decreased linearly with RPM. The UN did not change but total manure N decreased linearly with RPM (*p* = 0.054). The RPM (19.3 g/d) seemed to help cows overcome the metabolizable Met deficiency while mitigating manure N excretions to the environment.

## 1. Introduction

Methionine (Met) is one of the most limiting amino acids for lactating dairy cows in the US [1]. When supply to the mammary glands is limited, a single EAA, such as Met decreases milk protein synthesis and makes the supply of the other AA surplus relative to the requirement. The liver catabolizes surplus AA and converts the N into urea, a large proportion of which is excreted in the urine. Moreover, the data of non-ruminant species highlight that dietary Met deficiencies can decrease the digestion of proteins in the small intestine and thus increase fecal N losses [2,3,4]. Once excreted into the environment, most of the urinary N is volatilized readily to ammonia that contributes to air and water pollution [5]. During manure storage, fecal N is transformed gradually to ammonia, nitrate, and nitrous oxide that contribute to air and water pollution, and global warming [6]. Under current feeding conditions, dairy cows convert only 25–30% dietary crude protein into milk protein while the balance is excreted in urine and feces [7,8]. In addition to affecting adversely the environment, dietary N lost in manure represent a significant economic loss to dairy producers as protein is the most expensive nutrient in the diet [9]. Therefore, feeding to alleviate dietary EAA deficiencies and enhance EAA supply to the udder is critical in improving the economic and environmental sustainability of dairy production [10].

A typical lactating cow diet in the US is based on corn and soybean and supplies 25% less metabolizable Met than the requirement predicted by current nutrient requirement models [11]. Therefore, formulating diets with rumen-protected Met is critical to capitalize on the maximum production potential of modern dairy cows. The encapsulation is considered the most successful technology for producing rumen-protected nutrients [12]. Microencapsulation encompasses a group of encapsulation technologies producing small particles called microcapsules where the nutrient is covered with multiple layers of coating materials that are resistant to ruminal degradation, but degraded in the post-ruminal environment [13]. Lipid-based coating materials are widely used for encapsulation because they are less expensive, and the use can be justified easily as it is a regular component of the diet [14]. Microencapsulated fatty acids from linseed oil have been shown to increase omega-3-fatty acid concentration in cow milk fat [15] and intramuscular fat of steers [16] suggesting high bioavailability. Although dietary supplementations of microencapsulated Met have been shown to increase milk yield of dairy cows [17], the impact on bioavailability (e.g., plasma Met concentrations), milk protein efficiency, or environmental sustainability is poorly understood. The study objective was to determine the effects of rumen-protected Met by microencapsulation (RPM; Timet^®^, Vetagro S.p.A, Reggio Emilia, Italy) on AA supply to the udder, milk production, and manure N losses of high producing dairy cows fed a corn and soybean-based diet.

## 2. Materials and Methods

### 2.1. Animals, Experimental Design, and Treatments

All procedures were approved by the Institutional Animal Care and Use Committee (IACUC: 18-091) of Iowa State University (Ames, IA, USA). Forty-eight multiparous Holstein cows [127 ± 41 days in milk (DIM) and 671 ± 8 kg BW; mean ± SD at the beginning of the trial] were used in replicated 4 × 4 Latin square design including four treatments and four 28 d periods. In each period, cows within a square were assigned randomly to one of four dietary treatments including a total mixed ration (TMR) serving as the control diet (CON), and CON supplemented with 20 (LM), 35 (MM), or 50 (HM) g/cow per day of microencapsulated DL-Met (Vetagro S.p.A, Reggio Emilia, Italy) providing 11.0, 19.3, and 27.5 g of Met to the diet, respectively. The CON was related to a predicted [18] metabolizable Met supply at 1.8% of metabolizable protein (MP, Table 1). The treatment sequences of individual cows across periods were determined at the beginning of the study to avoid carry-over effects. Cows were housed in a free-stall barn bedded with sand and equipped with feeding gates for individual cows (Calan Broadbent Feeding System; American Calan, Northwood, NH, USA) and free access to drinking water. Cows were fed individually an *ad libitum* amount of TMR (110% of previous day intake), which was delivered once daily (0700 h). Each RPM dose mixed in 100 g of ground corn was top-dressed separately on the basal diet of individual cows immediately after the diet was delivered in the morning. Feed allowance and refusals were recorded daily. Cows were milked three times per day at 0400, 1200, and 2000 h.

### 2.2. Sampling and Data Collection

#### 2.2.1. Feed Analyses

Samples of the basal TMR were collected from individual cow bins on day 27 and 28 of each period and pooled across cows and the days to obtain a composite sample for each period. Pooled TMR samples were placed in a forced-air oven at 65 °C for 48 h to determine DM, subsequently ground (1-mm screen; Wiley Mill, Arthur H. Thomas Co.; Philadelphia, PA, USA) and stored at room temperature until being sent for analyses of the nutrient composition in an external laboratory (Cumberland Valley Analytical Services; Waynesboro, PA, USA). Analyses included DM (method 930.15) [19], starch [20], NDF and ADF [21], and ash (method 942.05) [19]. The Kjeldahl method was used to determine the CP concentration of the diet.

#### 2.2.2. Production Performance

Milk yields of individual cows were recorded daily (*n* = 48). The milk yields during the last 7 d of each period were used to evaluate treatment effects in the statistical analysis described below. A sample of milk was obtained for each cow at each milking on the last two days (day 27 and 28) of each period. Milk samples of individual cows were composited proportionate to the milk yield on each day and stored at 4 °C with a preservative (Bronopol tablet; D & F Control System, San Ramon, CA, USA) until being analyzed. The milk samples were analyzed for true protein, fat, lactose, milk urea nitrogen (MUN), and somatic cell count (SSC) in Dairy Lab Services (Dubuque, IA, USA) using an infrared analyzer and procedures approved by AOAC International [22]. Milk yields on day 27 and 28 were used along with corresponding milk component concentrations to calculate milk component yields. Body weight and body condition score (BCS, 1 to 5 scale) were recorded for individual cows returning from morning milking (before feeding) on day 27 and 28 of each period. Wildman et al. [23] describe the scoring method. Two separate evaluators assessed BCS on those two days and the scores were averaged across the days.

#### 2.2.3. Blood Sampling and Animal Measurements

Blood was drawn from the coccygeal vessels and subcutaneous abdominal vein into 10 mL vacuum tubes with K2EDTA (BD, Franklin Lakes, NJ, USA) after each milking (400, 1200, and 2000 h) on day 27 and 28 in each period. Blood was collected only from eight cows having highest milk yield (≥40.0 kg/d) based on the records of the first period. Cantalapiedra-Hijar et al. [24] obtained statistically significant differences in arteriovenous differences of EAA and mammmary blood flow for two levels of metabolizable protein supply using only 4 cows in a 4 × 4 Latin square design. Therefore, we assumed to obtain a reasonable statistical power with 8 cows in the present study. Plasma was harvested by centrifuging the blood at 1500× *g* and 4 °C for 15 min and subsequently stored at −20 °C until analysis. The plasma of coccygeal and subcutaneous abdominal vein samples were composited separately for each cow in each period and analyzed for free amino acid concentrations (both EAA and non-essential AA) at the Agricultural Experiment Station Chemical Laboratories of the University of Missouri (Columbia, MO, USA). The analysis was performed with norleucine as the internal standard and cation-exchange chromatography (cIEC-HPLC) following the procedures of Deyl et al. [25] and Fekkes [26].

#### 2.2.4. Fecal and Urine Analyses

Feces and urine samples were collected from the same cows providing the blood (*n* = 8). Fecal samples were collected directly from the rectum at each milking (400, 1200, and 2000 h) on day 27 and 28 and composited to obtain one sample for an individual cow in each period. Indigestible neutral detergent fiber (iNDF) was used as an internal marker to estimate fecal output. Fecal samples were dried, and ground as described above for TMR samples. Dried and ground fecal samples and TMR samples of individual cow in each period were analyzed for DM (method 930.15) [19]. The iNDF content was determined in quadruplicate by incubating 5 × 10 cm Dacron bags containing 1.25 g of TMR or feces in the rumen of two rumen-cannulated cows for 288 h [27]. The bags were retrieved from the rumen, washed, dried, and analyzed for NDF [21]. Spot urine samples were collected at each milking (400, 1200, and 2000 h) on day 27 and 28 of each period. Samples were placed in ice immediately after collection and frozen at −20 °C until being analyzed. The frozen urine samples of individual cows were thawed in ice and composited across the days of each period before being analyzed for creatinine concentration in the Department of Veterinary Pathology at Iowa State University (Ames, IA, USA). The composited feces (dried and ground through a 1.0 mm screen) and urine samples were analyzed also for total N content using the Kjeldahl digestion (AQ300 Discrete Analyzer, Seal Analytical, Inc. Mequon, WI, USA).

### 2.3. Calculations and Statistical Analysis

#### 2.3.1. Calculations

Energy corrected milk (ECM, kg/d) was calculated using the following quation [28].

ECM=[0.327×milk yield, kg/d]+[12.95×milk fat, kg/d]+[7.65×milk protein, kg/d]

Fecal DM output (kg/d) was calculated with DMI (kg/d) and iNDF concentration (fraction of DM) of TMR (iNDF_TMR_) and feces (iNDF_FEC_) using the following equation.

$$\mathrm{Fecal}\;\mathrm{DM}\;\mathrm{output}=\frac{{\mathrm{iNDF}}_{\mathrm{TMR}}\times \mathrm{DMI}}{{\mathrm{iNDF}}_{\mathrm{FEC}}}$$

Urine volume (L/d) was calculated using creatinine concentration in urine (CREAT_c_, mg/L) and BW (kg) assuming 29.0 mg of creatinine is excreted in urine for every kg of BW [29].

$$\mathrm{Urine}\;\mathrm{volume}\;=\frac{\mathrm{BW}\;\times 29}{{\mathrm{CREAT}}_{c}}\;$$

Fecal and urinary N output (g/d) were calculated by multiplying fecal DM output and urine volume by the total N concentration in feces and urine, respectively. Mammary plasma flow (L/h) was calculated by the Fick principle [30] using milk output of phenylalanine plus tyrosine (Milk_Phe+Tyr_, moles/d) and arteriovenous difference (AVd, AVd = arterial concentration—venous concentration) of phenylalanine plus tyrosine (AVd_Phe+Tyr_, moles/L) as shown in the following equation. The Milk_Phe+Tyr_ (moles/d) was calculated assuming phenylalanine plus tyrosine makes 11% of the true milk protein yield [24]. Moreover, Milk_Phe+Tyr_ was corrected for blood-born true protein secreted to milk with a correction factor of 3.5% [30]. Free phenylalanine and tyrosine in milk were assumed negligible.

$$\mathrm{Mammary}\;\mathrm{plasma}\;\mathrm{flow}=\left[\frac{{\mathrm{Milk}}_{\mathrm{Phe}+\mathrm{Tyr}}\times 0.965}{{\mathrm{AVd}}_{\mathrm{Phe}+\mathrm{Tyr}}}\right]\times \frac{1}{24}$$

Mammary net uptake of EAA (mmol/h) was calculated by multiplying the AVd (mmol/L) by mammary plasma flow (L/h).

Mammary net uptake of EAA=AVd×Mammary blood flow

The EAA in milk protein (g/d) to mammary uptake of EAA (g/d) ratio (EAA-milk: EAA-uptake) was calculated assuming EAA make 50% of milk protein yield [24,31]. Milk protein efficiency (MPE) was calculated by expressing milk true protein yield (MPY, kg/d) as a percentage of dietary CP intake (kg/d) where CP intake included CP from feed and rumen-protected Met.

$$\mathrm{MPE}=\frac{\mathrm{MPY}\;}{\mathrm{CP}\;\mathrm{intake}\;}\times 100$$

#### 2.3.2. Statistical Analysis

Data were analyzed with the MIXED procedure of SAS (Version 9.4, SAS Institute Inc., Cary, NC, USA) using the following model.

Y_ijkl_ = μ + T_i_ + S_j_ + P(S)_jk_ + C(S)_jl_ + ε_ijkl_,

where Y_ijkl_ = the response variable of interest, μ = the overall mean, T_i_ = the fixed effect of the ith treatment (i = CON, LM, MM, and HM), S_j_ = the fixed effect of the jth square (j = 1, 2, 3, and 4), P(S)_jk_ = the fixed effect of the kth period (k = 1, 2, 3, and 4) nested in jth square, C(S)_jl_ = the random effect of lth cow nested in jth square, and ε_ijkl_ = the random error assumed to be independent and identically distributed from a normal distribution with a mean of 0 and a variance of σ^2^ [~N (0, Iσ^2^)]. In conjunction with above model, the linear, quadratic, and cubic contrasts for the relationships of RPM dose with each response variable were analyzed. The contrast coefficients were obtained using the IML procedure of SAS. Statistical significance of the treatment effects and the contrasts was declared at *p* ≤ 0.05. The tendencies were declared at 0.05 < *p* ≤ 0.10.

## 3. Results and Discussion

In this study, we supplemented graded levels of rumen-protected Met by encapsulation (RPM) to a corn and soybean meal-based diet of high producing cows. The objective was to determine the impact on milk production performance, AA supply to and uptake by the mammary glands, and manure N excretions. The RPM contains DL-Met (55%) and lipids (40%). Therefore, 20.0 (LM), 35.0 (MM), or 50.0 (HM) g/d doses provided 11.0, 19.3, and 27.5 g of Met, and 8.0, 14.0, and 20.0 g of lipids to the basal diet (CON), respectively. The predicted [18] MP supply of CON was 2520 g/d, which was 96% of the predicted MP requirement (Table 1). The CON was predicted [18] to have a metabolizable Met flow of 46 g/d (1.8% of MP) and a metabolizable lysine flow of 166 g/d (6.6% of MP, Table 1). Consequently, metabolizable Lys: Met in the basal diet was 3.65 (Table 1). Assuming that 50% of Met in RPM is absorbed, metabolizable lysine: Met of LM, MM, and HM were calculated to be 3.2, 3.0, and 2.8, respectively, (data not shown) showing a linear decline. The basal diet fulfilled 98.4% of the energy requirement of lactation (Table 1). However, the lipids in RPM caused negligible changes to the energy supply because the predicted [18] net energy of lactation of HM was only 0.3% greater than that of CON (37.3 and 37.2 Mcal/d, data not shown). The above information, as well as the fact that a subsample of cows (8 vs. 48) provided data for blood and manure N analysis, would help better understand the results presented and discussed below.

### 3.1. Production Performance

The means and the relationships of DMI, milk yield, and milk components with RPM dose (*n* = 48) are presented in Table 2. Dry matter intake was similar at 23.3 kg/d across RPM doses (*p* = 0.388). Milk yield had a linear relationship with RPM dose (*p* = 0.026) and increased from 38.1 kg/d for CON to 39.7 kg/d for HM (*p* = 0.019). Mordenti et al. [17] also observed a milk yield increase in dairy cows (31.8 vs. 31.0 kg/d) for supplementation of 50 g/d microencapsulated Met containing 25 g of DL-Met. Zanton et al. [32] summarized the results of several studies through a meta-analysis and concluded non-significant effects of rumen-protected Met on DMI, as well as milk yield. However, that meta-analysis was focused on two other rumen-protected Met products. Moreover, the average metabolizable Met in the basal diet of Zanton et al. [32] was greater than that in the present study (2.0 vs. 1.8% of MP). Perhaps, the basal Met deficiency was not severe enough to capture a significant milk yield response in Zanton et al. [32]. The RPM dose had simply a numerically positive relationship with milk protein percentage (*p* = 0.113). Nonetheless, milk protein yield responded strongly to RPM dose (*p* = 0.004, Table 2). Relative to CON, milk protein yield did not change for LM but increased by 50 g/d (*p* = 0.037) for MM providing 19.3 g/d of Met to the diet. The current National Research Council model [18] recommends a metabolizable Met supply at 2.2% of MP to meet the Met requirement of lactating dairy cows [33]. The metabolizable Met supply of CON (1.8% of MP) had, however, a 10 g/d deficiency to meet that 2.2% requirement. The milk protein yield not increasing further for HM relative to MM (*p* = 0.541) suggests that MM might have corrected the deficiency and helped cows meet the Met requirement. Milk fat and lactose percentages did not change with RPM supplementation (*p* > 0.370). Fat yield did not also have a relationship with the RPM dose (*p* ≥ 0.193). On the other hand, lactose yield tended to increase linearly with it (*p* = 0.080, Table 2). Moreover, milk protein yield had a stronger relationship with lactose yield than milk fat yield (r = 0.92 vs. 0.72, data not shown). Perhaps, the metabolizable Met enhances milk protein synthesis and lactose synthesis using similar mechanisms [34]. In line with milk yield and milk protein yield, ECM increased linearly with RPM dose (*p* = 0.038). The ECM increased by 1.57 kg/d from CON to HM representing a 4% increase (*p* = 0.037). Milk urea nitrogen concentration remained unchanged at 13.54 mg/dL highlighting that whole-body N catabolism and urinary N output [35] were unaffected by the RPM supplementation. The impact of RPM on urinary N output will be discussed further in the following sections. Body weight or BCS did not change with RPM dose (*p* ≥ 0.404) indicating no effect of metabolizable Met supply on the mobilization of fat (e.g., subcutaneous), the mobilization of protein (e.g., skeletal muscle), or the net effect of those two processes on BW and BCS.

### 3.2. Arterial Concentrations, A–V Differences, and Mammary Uptake of EAA

Arterial plasma concentrations of EAA presented in Table 3 are concentrations in the blood from coccygeal vessels (*n* = 8). We assumed the coccygeal blood would be a reasonable alternative to external pubic arterial blood for studying the arterial EAA concentrations, AVd, and mammary EAA uptake of lactating dairy cows as demonstrated by Zhang et al. [36]. The arterial concentration of Met increased linearly with RPM dose (*p* < 0.001). The LM and HM providing 11.0 and 27.5 g/d of Met to the diet was associated with a 14 and 34% increase in arterial Met relative to CON, respectively (*p* ≤ 0.019). Because the availability of free AA in the small intestine can positively influence the blood concentrations [37,38], arterial Met concentration can be used as a tool to evaluate the duodenal flows of Met in dairy cows [39]. Therefore, the arterial Met increasing linearly with RPM supplementation could highlight successful rumen protection of Met, successful intestinal release of Met, or both by the present RPM product. The arterial concentration of lysine, tryptophan, and arginine increased linearly (*p* < 0.050), whereas branched-chain amino acid concentration remained unchanged with increasing RPM dose. Although the reason is obscure, Zhou et al. [40] also observed significant increments specifically in plasma concentrations of lysine, tryptophan, and arginine for a rumen-protected Met supplementation in dairy cows. Nonetheless, the RPM supplementation did not affect the total plasma EAA concentration calculated by excluding the Met concentration (Table 3).

The arterio-venous differences (AVd), net mammary uptake, and mammary plasma flow are presented in Table 4. The AVd of Met did not change with RPM dose (*p* > 0.230) indicating an increased utilization of arterial Met by the mammary glands (e.g., milk protein synthesis) in response to the RPM supplementation. However, net mammary uptake of Met increased linearly with RPM dose (*p* = 0.028) likely because of mammary plasma flow that linearly increased with RPM dose (*p* = 0.043). The literature does not provide much information on relationships between blood flow and graded Met supplementations. Guinard and Rulquin [41] report a quadratic decrease in mammary blood flow for duodenal infusions of Met (0, 8, 16, and 32 g/d), while Berthiaume et al. [42] did not see mammary plasma flow changing for the supplementation of rumen-protected Met at 0, 36, and 72 g/d in lactating dairy cows. Nonetheless, the linear increase in mammary plasma flow could be linked to plasma arginine increasing with RPM dose (Table 3) as arginine serves as a precursor in the biosynthesis of nitric oxide that is shown to enhance mammary blood flow of lactating goats [43]. The RPM supplementation did not affect AVd of the other EAA except valine (*p* = 0.035) and tryptophan (*p* = 0.027) that decreased linearly with the RPM dose. However, net mammary uptake of valine or tryptophan was not affected by RPM dose possibly because the uptake was also a function of mammary plasma flow that increased with RPM dose. Signifying further the impact of plasma flow, net mammary uptake of lysine, phenylalanine, and arginine increased linearly (*p* ≤ 0.045) even though AVd did not change with the RPM dose (*p* > 0.155). Net mammary uptake of Lys and Arg were not correlated with arterial concentrations (*p* > 0.240, data not shown) but the plasma flow (r > 0.62, *p* < 0.001, data not shown) highlighting the significance of blood flow in supplying precursors for the synthesis of milk. Although a linear decrease in metabolizable lysine: Met could be anticipated with increasing RPM supplementation, the ratio between the mammary uptake of lysine and Met remained similar at 3.9 across the treatments (*p* = 0.080, data not shown) suggesting a marginal impact of the differences in the ratio between absorbed Met and Lys at mammary gland level. The net mammary uptake of EAA increased for LM compared to CON (*p* = 0.012), decreased for MM compared to LM (*p* = 0.051), and increased again for HM compared to CON (*p* = 0.051) exhibiting a cubic relationship with RPM dose. The net mammary uptake represents the utilization of EAA for milk protein synthesis, tissue protein accretion, and catabolism [44]. The EAA-milk:EAA-uptake ratio reflects the efficiency of utilizing net EAA uptake for milk protein synthesis. The ratio decreased linearly (*p* = 0.029) with RPM dose indicating a decreased AA utilization efficiency for milk protein synthesis when AA supply (e.g., blood flow) increased. Similarly, Nichols et al. [31] observed that EAA-milk: EAA-uptake ratio decreased from 0.86 to 0.68 for abomasal infusion of 0.84 or 1.13 kg of EAA that increased net mammary uptake of EAA by 63% and milk protein yield by 30%. Moreover, this negative relationship between the AA utilization efficiency and RPM dose could explain to some extent the weak relationship between milk protein concentration and RPM dose [45] albeit milk protein yield increased significantly with RPM dose (Table 2).

### 3.3. Nitrogen Partitioning and Nitrogen Balance

Nitrogen outputs in feces, urine, and milk, and N balance are presented in Table 5. Those results correspond to the subset of cows (*n* = 8) providing AA data discussed above. Similar to DMI (Table 2), N intake did not change with RPM dose (*p* > 0.310). Fecal N output (g/d) tended to decrease linearly with the RPM dose (*p* = 0.081). The linear decrease in fecal N became more significant when it was expressed as a percentage of N intake (*p* = 0.020) highlighting an improvement in apparent total tract digestibility of dietary CP for the RPM supplementation. The literature provides limited data for relationships between the CP digestibility of dairy cows and graded levels of dietary RPM. In previous studies, the CP digestibility has been assessed very often for a single dose of RPM and shown to be unaffected. Nonetheless, the data on other animal species, such as rats, pigs, poultry, and fish, indicate Met supplementation can enhance the digestion and absorption of proteins by improving small intestinal morphology including the villus height [3,4], and stimulating the production of digestive enzymes in the pancreas and enhancing the activity of those enzymes [2,46]. Urine volume was similar at 47.7 L/d across treatment. Moreover, urinary N output (g/d or % of N intake) did not change with the RPM dose (*p* > 0.235). It is fair to acknowledge the lack of representativeness of urine volumes (L/d) and N outputs (g/d) determined with creatinine concentrations of spot urine samples as highlighted in Lee et al. [47]. However, Lee et al. [47] concluded also that such data could be still useful in discussing the treatment differences and trends. Owing to the significantly decreased fecal N output, total manure (feces + urine) N output (g/d) tended to decrease linearly with RPM dose (*p* = 0.054). The linear decrease in manure N output became further prominent (*p* = 0.006) when it was expressed as a percentage of N intake. Manure N decreasing relative to N intake represents an increased efficiency of the utilization of dietary protein, which is an expensive nutrient accounting for 42% of the total dietary nutrient cost of dairy cows [9]. Reductions in manure N excretions also improve the environmental sustainability of dairy farms as the transformation of manure N to ammonia, nitrate, and nitrous oxide can pollutes air and water and contribute to global warming [5,6]. Similar to the performance observed with all 48 cows (Table 2), the milk protein yield of those eight highest producing cows also increased linearly with RPM dose (*p* = 0.015). Milk protein efficiency (MPE) expressing milk protein yield as a percentage of dietary crude protein intake had a positive but quadratic relationship with RPM dose (*p* = 0.043, Table 5). The MPE tended to increase from CON to LM by 4.0 percentage units (34.3 vs. 30.3%, *p* = 0.077) while MPE of MM also remained numerically greater than that of CON (33.5 vs. 30.3%, *p* = 0.137). According to White [48], a 3.0–4.0 percentage unit increase in MPE could be related to about a 5.0% decrease in land use, water use, and greenhouse gas emissions of US dairy production. The N balance denoting the difference between N intake and manure and milk N outputs increased linearly with RPM dose (*p* = 0.009). Cows without RPM were related to a negative N balance of 71.8 g/d that decreased to 4.6 g/d for MM (*p* = 0.011). Considering together the milk protein yield (Table 2 and Table 5) and the N balance (Table 5) responses to RPM dose, cows seem to be able to meet the metabolizable Met requirement at MM.

## 4. Conclusions

Supplementation of microencapsulated DL-Met (0, 11.0, 19.3, and 27.5 g/d) to a diet deficient in metabolizable Met (~10 g/d) increased linearly milk yield, milk protein yield, and energy corrected milk yield without affecting DMI. Milk protein yield increased by 50 g/d for the dose providing 19.3 g/d of Met to the diet but the rate of the increments declined for further supplementation of RPM. Arterial Met, arginine, lysine, tryptophan, and plasma flow to the udder increased linearly with RPM dose. Net mammary uptake of EAA increased linearly with RPM dose possibly due to the increased mammary plasma flow because the AVd did not respond significantly to RPM dose. The RPM dose decreased linearly the percentage of dietary N excreted in feces indicating an improved apparent total tract digestibility of dietary crude protein. Despite the unaffected urinary N output, manure (feces + urine) N output decreased linearly while milk protein efficiency increased quadratically with RPM dose. Cows without RPM had a negative N balance, which started to become positive at RPM dose providing 19.3 g/d of Met to the diet. Overall, the present rumen-protected Met product providing 19.3 g/d of Met to the diet seems to help cows overcome the metabolizable Met deficiency of about 10 g/d.

## Figures and Tables

**Table 1:** Ingredient and analyzed chemical composition of basal diet with predicted nutrient supply ^1^.

Dietary Ingredient/Nutrient	Quantity
Diet composition (% of DM)	
Corn silage	40.3
Alfalfa hay	15.5
Ground corn	15.3
Soybean meal	9.3
Cottonseed	5.7
Quality Liquid Feed (Molasses)	2.9
Grain mix ^2^	11.0
Nutrient composition (% of DM)	
CP	17.3
NDF	31.8
ADF	21.5
Starch	23.7
Ash	8.1
Nutrient supply (NRC, 2001) ^3^	
NE_L_ (Mcal/d)	37.2
MP (g/d)	2520
Metabolizable Met (% of MP)	1.80
Metabolizable Lys (% of MP)	6.57
Metabolizable Lys: Met	3.65
Nutrient requirement (NRC, 2001) ^3^	
NE_L_ (Mcal/d)	37.8
MP (g/d)	2626

^1^ The averages of composite samples collected throughout the trial (*n* = 4). ^2^ DDG (43%), bloodmeal (11%), soybean hulls (10%), pork meat and bone meal (9%), sodium bicarb (7.4%), calcium carbonate (6%), choice white grease (5%), salt (3.1%), urea (2%), vitamin mix (2.3%), magnesium oxide (2.7%), monocalcium phosphate (1.4%), monensin (0.1), biotin and organic Zn (0.1%). ^3^ Determined using the data of control.

**Table 2:** Production performances, body weight, and body condition score (BCS) of lactating dairy cows (*n* = 48).

	Dietary Treatment ^1^				SEM	*p*-Value		
	CON	LM	MM	HM		Linear	Quadratic	Cubic
Dry matter intake, kg/day	23.59	23.39	23.02	23.33	0.46	0.388	0.488	0.484
Milk yield, kg/day	38.05 ^b^	39.31 ^ab^	39.16 ^ab^	39.73 ^a^	0.86	0.026	0.537	0.411
Milk protein, %	3.17	3.16	3.22	3.21	0.04	0.113	0.658	0.151
Milk protein, kg/day	1.20 ^b^	1.23 ^ab^	1.25 ^a^	1.26 ^a^	0.02	0.004	0.792	0.950
Milk fat, %	3.72	3.69	3.70	3.66	0.88	0.420	0.869	0.666
Milk fat, kg/day	1.40	1.44	1.44	1.44	0.04	0.193	0.445	0.705
Lactose, %	4.66	4.63	4.67	4.62	0.03	0.372	0.697	0.131
Lactose, kg/day	1.78	1.83	1.83	1.84	0.04	0.080	0.508	0.770
ECM, kg/d ^2^	39.73 ^b^	40.87 ^ab^	40.96 ^ab^	41.30 ^a^	0.89	0.038	0.501	0.670
MUN ^3^, mg/dL	13.58	13.55	13.71	13.32	0.23	0.413	0.384	0.493
SCC, ×10^3^/mL ^4^	281	394	223	389	149	0.730	0.534	0.686
Body weight, kg	672.8	671.5	670	671.5	8.5	0.545	0.688	0.998
BCS	3.34	3.33	3.32	3.32	0.03	0.404	0.488	0.484

^1^ CON = Control (0 g/d Timet^®^); LM = Low Met (20 g/d Timet^®^ providing 11 g/d of Met); MM = Medium Met (35 g/d Timet^®^ providing 19.3 g of Met); HM = High Met (50 g/d Timet^®^ providing 27.5 g of Met). ^2^ Energy-corrected milk, ^3^ Milk urea nitrogen, ^4^ Somatic cell count in milk, ^a,b^ Values in the same row with different superscript differ significantly (*p* < 0.050).

**Table 3:** Arterial plasma concentrations of essential amino acids (µmol/L) of the highest producing cows (*n* = 8).

	Dietary Treatment ^1^				SEM	*p*-Value		
	CON	LM	MM	HM		Linear	Quadratic	Cubic
Methionine	18.94 ^c^	21.66 ^b^	22.45 ^b^	25.33 ^a^	0.79	<0.001	0.655	0.289
Lysine	79.03	81.92	84.4	89.8	5.72	0.049	0.611	0.851
Phenylalanine	50.84	50.04	46.93	50.49	3.14	0.532	0.222	0.188
Isoleucine	125.8	130.7	120.9	128.2	9.9	0.915	0.913	0.148
Leucine	198.3	195.4	183.1	192.8	16.4	0.399	0.480	0.332
Valine	309.4	311.6	299.1	310.2	20.8	0.810	0.696	0.354
Threonine	123.2	120.5	126.0	131.7	9.83	0.064	0.137	0.632
Tryptophan	33.39 ^b^	35.71 ^a^	36.20 ^a^	37.34 ^a^	2.02	0.002	0.510	0.546
Histidine	44.73	47.48	45.63	50.09	2.04	0.089	0.620	0.197
Arginine	76.11	77.07	82.01	85.71	4.63	0.026	0.480	0.679
BCAA ^2^	633.5	637.9	603.2	631.5	47.5	0.668	0.652	0.278
EAA ^3^	1046.2	1052.3	1028.3	1077.3	52.4	0.619	0.469	0.414

^1^ CON = Control (0 g/d Timet^®^); LM = Low Met (20 g/d Timet^®^ providing 11 g/d of Met); MM = Medium Met (35 g/d Timet^®^ providing 19.3 g of Met); HM = High Met (50 g/d Timet^®^ providing 27.5 g of Met). ^2^ BCAA = isoleucine + leucine + valine, ^3^ EAA = the sum of all EAA except Met, ^a–c^ Values in the same row with different superscript differ significantly (*p* < 0.05).

**Table 4:** Arteriovenous (AV) difference, mammary plasma flow, and net mammary uptake of essential amino acids (EAA) of the highest producing cows (*n* = 8).

	Dietary Treatment ^1^				SEM	*p*-Value		
	CON	LM	MM	HM		Linear	Quadratic	Cubic
AV difference (µmol/L)								
Methionine	12.9	11.5	11.7	11.6	0.8	0.235	0.381	0.652
Lysine	49.3	45.6	45.3	45.7	3.6	0.362	0.484	0.891
Phenylalanine	22.5	19.7	19.5	19.9	1.5	0.156	0.270	0.851
Isoleucine	46.2	38.8	36.8	39.1	4.1	0.131	0.202	0.924
Leucine	74.4	61.6	59.5	62.4	6.2	0.101	0.172	0.943
Valine	69.2 ^a^	55.8 ^ab^	50.7 ^b^	53.2 ^ab^	5.8	0.035	0.195	0.881
Threonine	33.2	29.4	28.4	27.5	2.7	0.128	0.629	0.885
Tryptophan	4.22	4.20	3.09	2.98	0.6	0.027	0.700	0.274
Histidine	12.1	10.5	10.1	9.6	0.9	0.062	0.644	0.874
Arginine	35.7	33.1	30.2	33.0	2.8	0.249	0.273	0.478
BCAA ^2^	189.8	156.2	147.0	154.7	15.8	0.069	0.182	0.956
EAA ^3^	348.3	299.3	284.8	293.5	26.1	0.090	0.269	0.978
Plasma flow, L/h	740 ^b^	1017 ^a^	985 ^a^	992 ^a^	97	0.043	0.097	0.428
Net mammary uptake, mmol/h								
Methionine	9.5 ^b^	11.6 ^a^	11.0 ^ab^	11.4 ^ab^	0.7	0.028	0.071	0.143
Lysine	36.2 ^b^	46.2 ^a^	42.5 ^ab^	45.1 ^ab^	2.4	0.025	0.10	0.083
Phenylalanine	16.5 ^b^	20.2 ^a^	18.5 ^ab^	19.7 ^a^	1.1	0.027	0.089	0.036
Isoleucine	33.1	40.0	34.5	38.7	2.4	0.167	0.385	0.029
Leucine	53.6	63.8	55.9	61.9	3.9	0.175	0.175	0.036
Valine	49.1	58.2	47.2	53.4	3.4	0.798	0.493	0.023
Threonine	24.1	29.9	26.6	27.6	2.1	0.351	0.191	0.178
Tryptophan	2.8 ^b^	4.2 ^a^	2.9 ^b^	3.0 ^ab^	0.5	0.712	0.029	0.016
Histidine	10.1	11.9	10.7	11.0	0.8	0.545	0.210	0.178
Arginine	26.4 ^b^	33.3 ^a^	28.3 ^b^	32.7 ^ab^	1.9	0.045	0.310	0.011
BCAA ^2^	135.8 ^b^	162 ^a^	137.6 ^b^	154.0 ^ab^	9.3	0.325	0.412	0.025
EAA ^3^	251.9 ^b^	307.9 ^a^	267.1 ^b^	293.3 ^ab^	15.9	0.144	0.212	0.023
Net EAA uptake, g/d ^4^	702	1090	919	999	116	0.062	0.077	0.081
EAA in milk protein, g/d	655 ^b^	735 ^a^	715 ^a^	743 ^a^	38	0.015	0.202	0.158
EAA-milk: EAA-uptake ^5^	0.92 ^b^	0.68 ^a^	0.79 ^a^	0.74 ^a^	0.13	0.029	0.143	0.184

^1^ CON = Control (0 g/d Timet^®^); LM = Low Met (20 g/d Timet^®^ providing 11 g/d of Met); MM = Medium Met (35 g/d Timet^®^ providing 19.3 g of Met); HM = High Met (50 g/d Timet^®^ providing 27.5 g of Met), ^2^ isoleucine + leucine + valine, ^3^ the sum of all EAA excluding Met. ^4^ Net mammary uptake of all EAA including Met, ^5^ Ratio between EAA in milk protein (g/d) and net mammary uptake of EAA including Met, ^a,b^ Values in the same row with different superscript differ significantly (*p* < 0.05).

**Table 5:** Nitrogen (N) intake, N outputs, and N balance of the highest producing cows (*n* = 8) ^1^.

	Dietary Treatment ^2^				SEM	*p*-Value		
	CON	LM	MM	HM		Linear	Quadratic	Cubic
N intake, g/d	691.4	693.3	689.1	734.8	30.2	0.314	0.376	0.623
Feces								
DM, kg/d	11.39	9.86	10.23	9.95	1.03	0.319	0.509	0.611
N, g/d	315.6	253.1	247.6	230.8	37.9	0.081	0.528	0.722
N, % of N intake	44.8	36.8	35.7	31.4	4.1	0.020	0.687	0.602
Urine								
Creatinine, mg/dL	48.1	43.3	45.6	43.2	4.5	0.323	0.615	0.353
Urine output, L/d	42.7	47.9	48.2	51.9	5.4	0.069	0.842	0.559
N, g/d	238.1	241.6	213.4	222.8	20	0.402	0.982	0.404
N, % of N intake	34.3	34.9	31	30.5	2.8	0.240	0.733	0.509
Feces + Urine								
N, g/d	558.9	490.6	460.5	447.6	46.8	0.054	0.555	0.995
N, % of N intake	79.7 ^a^	71.1 ^ab^	66.6 ^ab^	61.2 ^b^	4.5	0.006	0.857	0.874
Milk								
True protein, g/d	1309 ^b^	1471 ^a^	1429 ^a^	1489 ^a^	76	0.015	0.202	0.158
N, g/d ^3^	205.2 ^b^	230.5 ^a^	224.1 ^a^	232.9 ^a^	11.9	0.015	0.202	0.158
MPE, % ^4^	30.3 ^a^	34.3 ^b^	33.5 ^ab^	33.0 ^ab^	2.1	0.109	0.043	0.399
N balance ^5^	−71.8 ^c^	−29.1 ^bc^	4.6 ^ab^	50.6 ^a^	35.8	0.009	0.779	0.924

^1^ The data correspond to the last two days (d 27 and 28) of each period, ^2^ CON = Control (0 g/d Timet^®^); LM = Low Met (20 g/d Timet^®^ providing 11 g/d of Met); MM = Medium Met (35 g/d Timet^®^ providing 19.3 g of Met); HM = High Met (50 g/d Timet^®^ providing 27.5 g of Met). ^3^ Milk true protein/6.38, ^4^ Milk protein efficiency = (milk true protein yield/CP intake) × 100, ^5^ N intake (g/d)—fecal N (g/d)—urinary N (g/d)—Milk true protein N (g/d), ^a–c^ Values in the same row with different superscript differ significantly.

## Data Availability

All datasets collected and analyzed during the current study are available from the corresponding author on fair request.
